# Were US Asian Indian decedents with atherosclerosis more likely to have concurrent diabetes mellitus? Analysis of national multiple cause of mortality data (2012–2019)

**DOI:** 10.1186/s13098-022-00933-7

**Published:** 2022-10-28

**Authors:** Deepak R. Nair, Abhyuday Chauhan, Dhananjay Vaidya

**Affiliations:** 1 Armed Forces Medical College, Pune, India; 2 Department of Medicine, Johns Hopkins University School of Medicine, 1830 E. Monument Street / Suite 8028, Baltimore, MD 21287-0003 USA

**Keywords:** Asian Indian, Mortality, Atherosclerosis, Diabetes

## Abstract

**Background:**

Asian Indians (AI) are at high risk for both atherosclerotic diseases (ATH) and diabetes mellitus (DM). We analyze the clustering of these two comorbidities as contributing causes of death in AI versus Non-AI populations in the US**.**

**Methods:**

Using Mortality Multiple Cause-of-Death Files (2012–2019) from the National Center for Health Statistics, we included deaths at age ≥ 45 years among US residents where AI versus Non-AI status could be ascertained (n = 55,461 AI; n = 20,090,038 Non-AI) and identsified ATH (ICD10: I20-I25, I63, I70) and DM (ICD10: E10-E14) as contributing causes of death. We calculated the tetrachoric correlation (Rho) between these contributing causes and the difference in the fraction of deaths involving DM in those with versus without ATH.

**Results:**

Among AI decedents, 29.9% of deaths included ATH as a contributing cause, 16.4% included DM as a contributing cause with 8.3% deaths being included in the overlap (Rho = 0.36, SE = 0.007) whereas, among Non-AI, 22.4% of deaths included ATH as a contributing cause, 10.0% included DM as a contributing cause with 4.1% deaths being included in the overlap (Rho = 0.31, SE = 0.001). Thus, DM and ATH as co-occurring causes correlated more strongly in AI versus Non-AI (p < 0.001). Further, this difference in clustering of DM with ATH was highest for younger AI women (age < 60 years) compared to comparable Non-AI women.

**Conclusions:**

The more frequent co-occurrence of DM and ATH as causes of death among AI compared to Non-AI suggest that the increased burden of these diseases among AI during life has vicious synergistic consequences in terms of mortality. Public health strategies targeted to AI should focus on prevention and clinical treatment of both conditions jointly, in all adults, and especially in women < 60 years.

**Supplementary Information:**

The online version contains supplementary material available at 10.1186/s13098-022-00933-7.

## Introduction

Individuals of Asian Indian (AI) national origin are the largest representative sample of South Asian ethnicity (India, Pakistan, Bangladesh, Nepal, Bhutan, and Sri Lanka) in the US [1]. AI have a higher burden of diabetes mellitus (DM) [2] in both native settings [3] and diaspora populations [4] and are more likely to succumb to DM complications in comparison to individuals of other national origins [5]. The unique cardiometabolic risk profile of this burgeoning ethnic population in the US has recently sparked much interest in the medical community [4–6].

Atherosclerotic—diseases (ATH) related events, predominantly coronary heart disease and cerebrovascular disease, are the leading cause of mortality in AI [7]. Although ATH- related mortality has dipped in the US over the past few years, AI have not enjoyed a proportional decrease [2, 8]. AI are also more likely to succumb to ATH prematurely compared to other racial/ ethnic groups [9]. While it is not fully established which of the conventional risk factors lend such disparity for excess ATH burden in AI [10–12], South Asian ethnicity itself has recently been recognized as a “risk modifier” for ATH [13]. It is second only to DM in ATH risk prediction [14].

DM confers an approximately two-fold increased risk of ATH-related morbidity and mortality relative to non-DM, more so among the younger ages [15]. Previous studies have shown that AI carry a much higher burden of DM and ATH than individuals of other national origins in the US (Non-AI) [2, 8, 16]. However, it is not known how strongly DM and ATH cluster as contributing causes of death in AI versus Non-AI. We use comprehensive US Vital Statistics Mortality Data to compare the co-occurrence of DM and ATH as causes of mortality in AI versus Non-AI.

## Methods

### Data

We used annual death records in the Mortality Multiple Cause-of-Death Files compiled by the National Center for Health Statistics (NCHS), which are available for public use*.* The information reported on death certificates completed by funeral directors, attending physicians, medical examiners, and coroners is used to create these datasets. Causes of death were coded according to the ICD10. We pooled data from 2012 to 2019 to create our final dataset. The reason for limiting our analysis to these eight years is that 2020 data were excluded due to the disproportionate number of deaths due to COVID-19. In addition, although disaggregated data for the Asian-American population has been reported in US Death Certificates since 2003, consistency in reporting decedents of AI descent by the participating US states improved only by 2012 [17].We also added data from 2003–2012 and repeated our analyses for sensitivity analyses but these are not reported in detail. As this dataset is publicly-available and de-identified, it was exempt from Institutional Review Board approval.

We used the following variables from the NCHS Mortality Multiple Cause-of-Death Files for analysis: Resident Status, Sex, Age Recode 12, Race, Hispanic Origin Recode, Number of Record-Axis Conditions, and Record-Axis Conditions (numbers 1 to 20). Each Record-Axis Condition indicates a contributing cause of death as recorded on the death certificate. Tape locations for these fields were identified with the help of the accompanying user guide.

### Study population

We restricted our analysis to age 45 years and older as ATH and DM as contributing causes of death are less likely at younger ages. We used five age groups in our analyses according to Age Recode 12 to define the mid-decadal age. They were: 45–54 years, 55–64 years, 65–74 years, 75–84 years, and 85 years and over. Death records of foreign residents or those with missing data on age were removed. Further, we recoded available data on Race and Hispanic—Origin to exclude death records with any missing data on race, ethnicity, or nationality. Next, we grouped AI decedents as we were primarily interested in understanding differences compared to decedents of Non-AI. The latter group comprised Non-Hispanic White, Non-Hispanic Black, American Indians, Hispanics, Japanese, Chinese, Filipino, Korean, Vietnamese, and Other Asian and Pacific Islanders. Thus, the final number of death records in our 2012–2019 dataset was 20,145,499, of which 55,461 (0.28%) were AI decedents.

### Definition of ATH and DM as contributing causes of death

We used ICD10 codes to identify ATH-related deaths (either of the following: ischemic heart disease, ischemic stroke, atherosclerosis; ICD10 range: I20-I25, I63, I70, respectively) and DM-related deaths (ICD10 range: E10-E14) in the AI and Non-AI groups. Any mention anywhere in the multiple causes of death records (Record Axis Conditions) of ATH and DM were included.

### Statistical analysis

All statistical analyses were implemented using R software, version 4.1 (R Project for Statistical Computing), with the polycor package for calculation of tetrachoric correlation (Rho) and its standard error [18]. We tabulated proportions of patterns of co-occurrence of DM and ATH by AI and Non-AI groups*.* To determine the association of DM and ATH beyond chance co-occurrence due to their prevalence, we calculated the Rho between DM and ATH separately in the AI and Non-AI groups*.* Rho describes the relation between two dichotomous variables and represents the correlation of underlying normally distributed latent variables which manifest as the dichotomous DM and ATH causes of death [19]. We further examined whether this association differed by age decade and sex and calculated the difference in the fraction of deaths with DM in those with ATH versus those without ATH as a co-occurring cause of death*.* The difference of Rho between AI and Non-AI was calculated and the null hypotheses of no difference was tested as a difference between two normal distributions (Additional file 1, data download instructions and R Markdown analysis script).

## Results

### Profile of the deceased

In this dataset of Mortality Multiple Cause-of-Death Files from 2012–2019, there were 4,518,524 (22.43%) ATH-related deaths and 2,017,221 (10.01%) DM-related deaths (Table 1).

**Table 1:** Proportions of patterns of co-occurrence of DM and ATH by AI and Non-AI groups

Asian Indian national origin status	The cluster of contributing causes of death	n (%) of death records associated with each cluster
AI	ATH & DM	4603 (8.29%)
	ATH & Non-DM	11,960 (21.56%)
	Non-ATH & DM	4505 (8.12%)
	Non-ATH & Non-DM	34,393 (62.01%)
Total number of deaths in AI		55,461
Non-AI	ATH & DM	826,262 (4.11%)
	ATH & Non-DM	3,675,699 (18.29%)
	Non-ATH & DM	1,181,851 (5.88%)
	Non-ATH & Non-DM	14,406,226 (71.71%)
Total number of deaths in Non-AI		20,090,038

AI indicates Asian Indian; Non-AI, Non-Asian Indian; ATH, Atherosclerotic disease defined as either of the following:—ischemic heart disease (ICD10 I20–I25), ischemic stroke (ICD10 I63), or atherosclerosis (ICD10 I70)

*DM* Diabetes Mellitus (ICD10 E10–E14), *Non-ATH* Non-Atherosclerotic disease, *Non-DM* Non-Diabetes Mellitus

### Correlation between ATH and DM as contributing causes of death

**Fig. 1:**
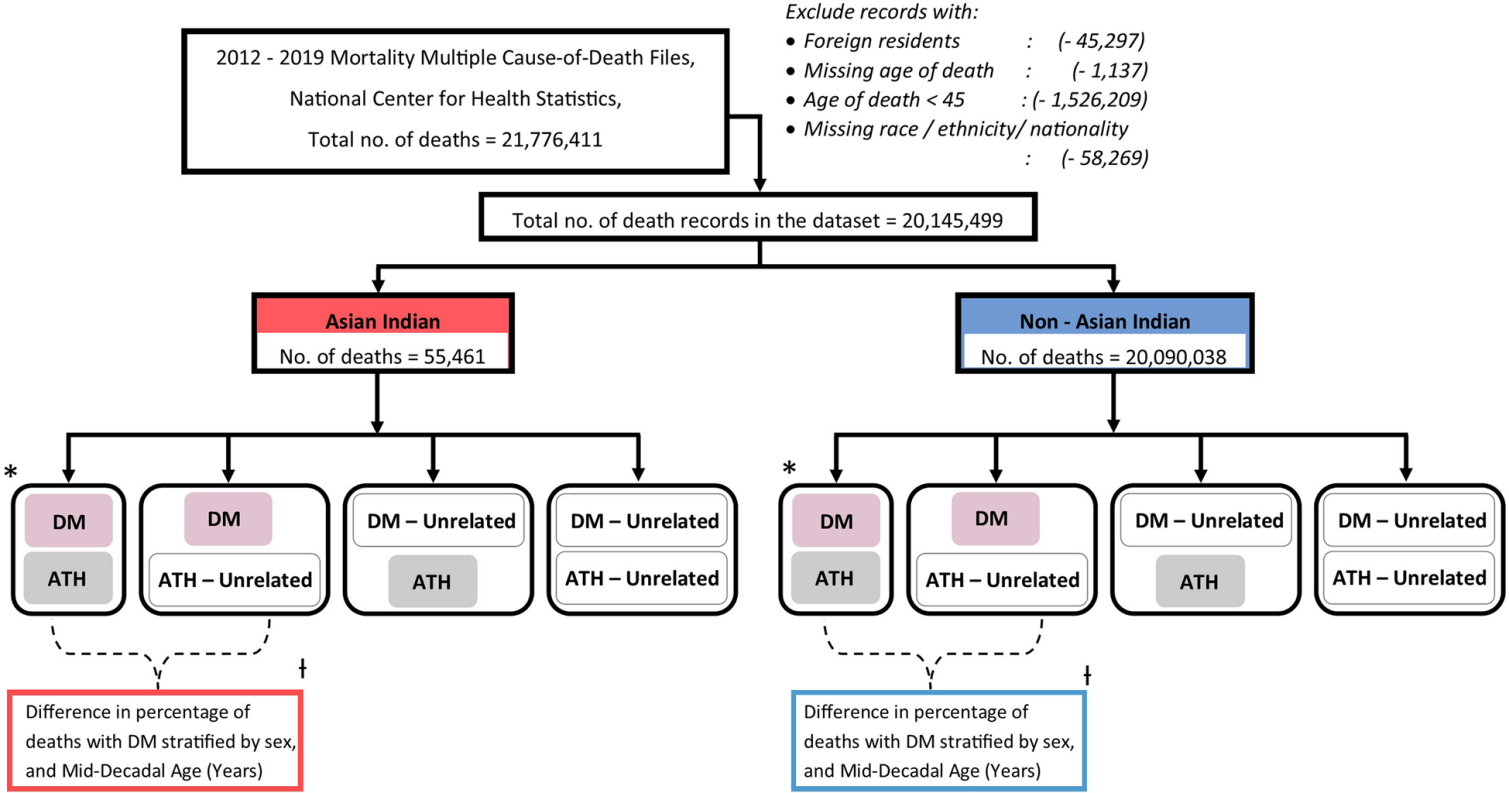
Flowchart showing study design and methodology. * Dichotomous tetrachoric correlation (Rho) between diabetes mellitus and atherosclerotic disease as co-occurring causes of death were identified in Asian Indian and Non-Asian Indian groups. + To examine whether this association (i.e., Rho) differed by age decade and sex, the difference in the fraction (% excess) of deaths with diabetes mellitus in those with atherosclerotic disease versus those without atherosclerotic disease, as a co-occurring cause of death, was calculated. Atherosclerotic disease (ATH): any of ischemic heart disease (ICD10 I20–I25), ischemic stroke (ICD10 I63), or atherosclerosis (ICD10 I70); diabetes mellitus (DM) (ICD10 E10–E14)

The number of ATH deaths when DM also contributed was 830,865 (18.4%). Among AI decedents, 29.9% of deaths included ATH as a contributing cause, 16.4% included DM as a contributing cause with 8.3% deaths being included in the overlap (Rho = 0.36, SE = 0.007) whereas, among Non-AI decedents, 22.4% of deaths included ATH as a contributing cause, 10.0% included DM as a contributing cause with 4.1% deaths being included in the overlap (Rho = 0.31, SE = 0.001) (difference between AI versus Non-AI p < 0.001) (Table 1, Fig. 1).

**Fig. 2:**
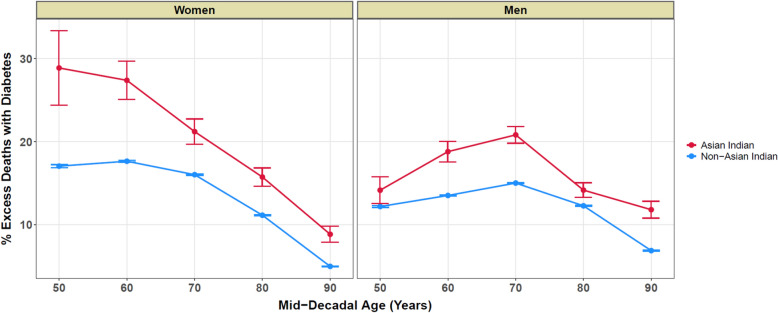
Excess diabetes mellitus-related deaths (in percentage) among decedents with and without atherosclerotic diseases—United States (2012–2019). The graph shows the difference in the percentage of deaths with diabetes stratified by sex, mid-decadal age (years), and Asian Indian national origin status

Table 2 and Fig. 2 show the excess fraction of deaths due to DM when ATH also contributed versus when ATH did not contribute in AI compared to Non-AI by age decade and sex. In comparison to Non-AI, the contribution of DM as a cause of death in ATH-related versus ATH-unrelated deaths was significant across all age groups in AI, both men and women. Notably, this excess number of DM deaths in deceased AI with ATH was mostly higher among women than men of the same age group, except for those died around 90 years of age; this difference was most apparent among those died at age 60 or earlier.

**Table 2:** Distribution of excess diabetes mellitus-related deaths among decedents with and without atherosclerotic diseases, United States, 2012–2019

Mid-decadal age (years)	Sex	ATH / Non-ATH status among DM-related deaths	Asian-Indian				Non-Asian Indian			
			*n*	Total	%	Excess DM in ATH—related deaths*	*n*	Total	%	Excess DM in ATH—related deaths*
50	M	ATH	185	800	23.13	14.18	31,298	162,254	19.29	12.21
		Non-ATH	186	2077	8.95		47,362	668,620	7.08	
	F	ATH	42	117	35.89	28.88	14,974	62,619	23.91	17.06
		Non-ATH	86	1226	7.01		32,377	472,850	6.85	
60	M	ATH	567	1796	31.57	18.82	88,178	404,792	21.78	13.54
		Non-ATH	460	3606	12.75		108,990	1,321,438	8.24	
	F	ATH	175	473	36.99	27.38	43,588	167,672	25.99	17.65
		Non-ATH	209	2176	9.61		79,029	947,476	8.34	
70	M	ATH	936	2812	33.29	20.83	141,083	581,588	24.26	15.03
		Non-ATH	651	5226	12.46		155,445	1,684,326	9.23	
	F	ATH	371	1098	33.79	21.22	75,301	300,274	25.08	16.04
		Non-ATH	477	3795	12.57		126,824	1,402,775	9.04	
80	M	ATH	910	3333	27.30	14.18	142,785	692,364	20.62	12.29
		Non-ATH	831	6332	13.12		160,073	1,920,996	8.33	
	F	ATH	609	2070	29.42	15.75	95,688	494,749	19.34	11.16
		Non-ATH	742	5429	13.67		165,945	2,027,871	8.18	
90	M	ATH	433	2055	21.07	11.82	94,296	711,185	13.26	6.90
		Non-ATH	371	4012	9.25		117,073	1,840,651	6.36	
	F	ATH	375	2009	18.67	8.87	99,071	924,464	10.72	5.00
		Non-ATH	492	5019	9.80		188,733	3,301,074	5.72	

ATH, Atherosclerotic disease defined as either of the following:—ischemic heart disease (ICD10 I20–I25), ischemic stroke (ICD10 I63), or atherosclerosis (ICD10 I70); DM, Diabetes Mellitus (ICD10 E10–E14)

*Non-ATH* Non-Atherosclerotic disease, *Non-DM*: Non-Diabetes Mellitus

*Calculated as follows: % deaths in (ATH&DM) cluster minus % deaths in (Non-ATH&DM) cluster, for each sex group per mid-decadal age group. The resulting data points are used to produce Fig. 2

In sensitivity analyses, adding data from the years 2003–2012 to our analyses did not alter our qualitative results.

## Discussion

In this study, we have shown that DM and ATH cluster as contributing causes of death more strongly in AI than Non-AI. We also found that the difference in the fraction of deaths with DM when ATH was a contributing cause relative to when ATH did not contribute was higher for both AI men and women across all age groups but more so among younger AI women (age ≤ 60 years). However, DM is rarely the primary cause of mortality; it is more likely to be an antecedent to vascular dysfunction, which can directly cause death [20]. In this context, our second finding, though only in mortality data, could be interpreted as follows: during life, DM as a risk factor for subsequent ATH and dual DM/ATH contribution to mortality is more salient in AI as compared to Non-AI. This contribution was significant for all age groups studied, both men and women. However, the excess contribution of DM in ATH-related deaths in AI was mainly higher among women than men of the same age group, and this difference was most apparent at age ≤ 60.

Our findings are in line with current literature that suggests the existence of excess dual burden of ATH and DM in AI versus Non-AI [2, 8, 16]. Further evidence regarding the subclinical disease during life comes from a recent study performed in the ongoing Mediators of Atherosclerosis in South Asians Living in America (MASALA) cohort. In this study, for any US race/ ethnic group with pre-existing diabetes free from cardiovascular disease at the time initial evaluation, the highest predicted probability for incident coronary artery calcium deposition, a marker of subclinical atherosclerosis, was observed in South Asians [21]. Our first finding takes this understanding further by quantitating the excess joint burden of ATH and DM as contributing causes of deaths in AI versus Non-AI.

Studies that have relied on electronic health records and health-system-based reports for data have reported a higher prevalence of DM and ATH in AI; however, these contrast with a recent observation by Satish et al*.,* who pooled data using self-reported questionnaires [22]. As several AI were potentially underdiagnosed owing to poorer access to healthcare and were, hence, unaware of their condition, Satish et al*.* found a significantly lower prevalence of DM and ATH in AI [22]. This observation is notable in the context of our findings as it highlights the need to step up the detection of DM and ATH in AI. Of note, the largest disparities due to poorer healthcare access to immigrants in the US are in the metabolic control of DM and ATH [23]. Poor healthcare access compounds the risk of undetected DM and ATH progression in AI, who are already genetically predisposed. In addition, their lower physical activity levels and culturally derived dietary practices further fuel the risk of developing these two cardiometabolic co-morbidities [8].

Our second finding is that the excess contribution of DM as a co-occurring cause of death in ATH-related versus ATH-unrelated deaths is most apparent in younger AI women (age ≤ 60). This finding is consistent with prior reports that the association between DM and mortality is generally higher in females and at younger ages [15, 24] and is most pronounced in AI women [2].

Our study has a major clinical implication in line with a recent observation by Coles et al*.* [25]. Whether DM itself incites the increased ATH mortality in AI or if it is the combined effect of the ‘Asian Indian phenotype’ (‘South Asian phenotype’) and DM is not presently known. Until future studies establish that association, our results indicate that public health strategies should focus on joint prevention and treatment of both ATH and DM in AI, especially in young adulthood and middle age. As suggested by the Emerging Risk Factors Collaboration, in those patients first diagnosed with DM, it is essential to prevent subsequent ATH and, conversely, to prevent DM in those who first develop ATH, because these diseases have multiplicative associations with mortality [24]. Further, our findings quantify the public health implication by quantitating at least a 4% excess co-occurrence of DM and ATH as contributing causes of death in AI versus Non-AI.

Our study has some limitations. Firstly, the data for our study is based on national death certificates, which may contain errors at the time of documentation. Secondly, we could not calculate the mortality rate using this dataset compiled by the NCHS as the national origin groups on US Death Certificates are not currently linked to census denominators. Therefore, we can only make indirect inferences about cause-specific rates observed in each subgroup using the cause-specific proportion of overall mortality in that subgroup as a proxy. As a next step to studying the mortality rate owing to concurrent ATH and DM as contributing causes in AI versus Non-AI, mortality data from US Death Certificates could be linked with US Census data. Nevertheless, our results add evidence to the growing field of study of cardiometabolic risk in the South Asian community.

Despite these limitations, our study has notable strengths. While previous studies have characterized mortality related to DM and ATH in Asian American populations using US death certificates [2, 8], to our knowledge, this is the first study to specifically examine DM and ATH clustering as contributing causes of death in AI versus Non-AI using the same mortality data. Our study findings also provide a more informed approach for physicians toward cardiometabolic disease prevention and health promotion in AI.

## Conclusion

In conclusion, we showed that AI carry an excess burden of ATH and DM clustering as contributing causes of death compared to the rest of the US population. We also showed that the clustering of DM with ATH was higher for both AI men and women across all age groups but more prominent among younger AI women (age ≤ 60 years). Results from future studies are needed to calculate mortality rate rather than mortality fraction in AI to verify and expand on our conclusion. Public health strategies should, therefore, focus on joint prevention and treatment of both ATH and DM in AI, especially in young adulthood and middle age.

## Supplementary Information

**Additional file 1.** Data download instructions and R markdown code (1) Data download instructions: pages 1-2 (2) R Markdown code for analysis: pages 3-28.

## Data Availability

The datasets analyzed during the current study are available in the Mortality Data repository, National Vital Statistics System, NCHS, CDC [https://www.cdc.gov/nchs/nvss/mortality_public_use_data.htm] (Accessed 25th August 2021). The R Language code used for analysis is available in the Additional File 1: Data Download Instructions and R Markdown Analysis Code.

## Abbreviations

AI: Asian Indian
Non-AI: Non-Asian Indian
ATH: Atherosclerotic disease(s)
DM: Diabetes Mellitus
