# Supplementary material for: Were US Asian Indian decedents with atherosclerosis more likely to have concurrent diabetes mellitus? Analysis of national multiple cause of mortality data (2012–2019)
Source: Diabetol Metab Syndr. 2022 Oct 28;14:159. doi: 10.1186/s13098-022-00933-7 (PMC9614193; doi:10.1186/s13098-022-00933-7)
Supplement: Supplementary file 1 — Additional file 1. Data download instructions and R markdown code (1) Data download instructions: pages 1-2 (2) R Markdown code for analysis: pages 3-28. [file 13098_2022_933_MOESM1_ESM.pdf]

Supplemental material for Publication:

Data Download Instructions and R Markdown Analysis Code

Data download instructions:

1. **Step 1**: Access the mortality files for each year (2012-2019) available at https://www.cdc.gov/nchs/nvss/mortality_public_use_data.htm (Accessed 25th August 2021). The same files can alternatively be accessed by going to CDC homepage and searching for "mortality files" in the search toolbox on the top right corner of the webpage. Please select the option "NVSS - Public Use Data File Documentation" from the search results.
2. **Step 2:** Download each of the eight years' files in its .DUSMCPUB file format along with the accompanying PDF User Guide. These files should be saved to a single folder in the working directory (e.g., Local Disk C) assigned by R Studio on the computer.
3. **Step 3:** Follow the instructions in the below-mentioned RMarkdown file to import, combine, and process these files in RStudio.
    a. **Tip:** The total folder size (containing eight mortality files) may be ~10GB and computer performance could slow down/ fail during analysis: if so, consider running the analysis online on RStudio using Cloud Computing Services - Amazon Web Services (AWS) by following these steps (Source:https://towardsdatascience.com/how-to-run-rstudio-on-aws-in-under-3-minutes-for-free-65f8d0b6ccda ; accessed 1st September 2021):
        i. Log in/ Create AWS account.
        ii. On the top right corner of AWS homepage, select the region you want your virtual machines to stay. Await confirmation by email to proceed further.
        iii. Scroll down on the Console Home webpage, click "Launch a Virtual Machine with EC2" under the option "Build a Solution".
        iv. Choose an Amazon Machine Image (AMI); Search for "rstudio server" → click search results from "Community AMIs" → Select RStudio
        v. Choose an Instance type: Select r5a with 128 GB memory.
        vi. Configure Instance: No changes
        vii. Add storage: No changes
        viii. Add tags: No changes
        ix. Configure a Security Group: Click "Add Rule" and add "HTTP" and "HTTPS". Set Source for all including SSH to "Anywhere". Click "Review and Launch".

x. Select to proceed without a key pair and launch instance. After instance has been launched, copy past the Public DNS (IPv4) to a new tab to open RStudio server. Username: “rstudio”; Password: (copy paste the Instance ID here).

xi. Upload the aforementioned folder containing all the mortality files in zipped format and run the R code.

xii. Remember to stop running the EC2 instance after analysis otherwise billing would continue.

US Asian Indians Dying with Atherosclerotic Disease are More Likely to have Concurrent Diabetes Mellitus

Deepak Rajendran Nair

06/April/2022

Contents

Summary 1
Preparations 1
Analysis 2
Import dataset 2
Preprocessing 6
Tidy data 6
Tetrachoric correlation (Rho): How strongly do contributing causes of atheroslerotic-disease(s) and diabetes mellitus **cluster** based on Asian Indian status? 13
% Excess deaths due to diabetes in atherosclerotic disease-related *vs.* unrelated deaths 17
Results 22
Graph 22
Check how the graph may appear to **color-blind** persons 24

Summary

Given that Asian Indians carry dual burden of atherosclerosis and diabetes mellitus, this study analyses how strongly the two co-morbidities cluster as contributing causes of deaths in Asian Indians compared to the rest of the US population. Death records from 2012-2019 were analysed in RStudio with the help of packages such as tidyverse (for data wrangling), polycor (for calculation of Rho), and ggthemes and colorBlindness add-on for ggplot2. The findings of this study indicate an urgent need to devise public health strategies that focus on prevention and clinical treatment of both co-morbidities jointly, in both Asian Indian men and women, especially during young adulthood and middle age.

Preparations

```
#Load packages
#Library readr (part of tidyverse package) reads the fixed width flat file
#"VSMORT.DUSMCPUB" (among others) & parses only relevant variables
if (!require(polycor)) {
  install.packages("polycor")
}
library(polycor)

#broom package contains augment and tidy function
if (!require(broom)) {
```

```r
  install.packages("broom")
}
library(broom)

#Packages for graph work
if (!require(ggthemes)) {
  install.packages("ggthemes")
}
if (!require(patchwork)) {
  install.packages("patchwork")
}
if (!require(colorBlindness)) {
  install.packages("colorBlindness")
}

library(ggthemes) #manual color scaling
library(patchwork) #stitching up 2 graphs into 1
library(colorBlindness)

# tidyverse package = ggplot2 + dplyr + tidyr + readr + purr + Others....
if (!require(tidyverse)) {
  install.packages("tidyverse")
}
library(tidyverse)

#purrr package contains map() function used to import multiple mort files in this analysis
library(purrr)

#fs package contains dir_ls function used to specify the folder in the working directory
#containing mort files
library(fs)

#Library readr (part of tidyverse package) reads the fixed width flat file
#"VSMORT.DUSMCPUB" (among others) & parses only relevant variables
library(readr)
library(dplyr)
```

Analysis

The 2012 - 2019 Mortality Multiple Cause Files used in this study was obtained from the National Center for Health Statistics, Centers for Disease Control and Prevention.

Import dataset

Only the relevant columns were parsed from the unzipped dataset **VSMORT.DUSMCPUB** file into RStudio. Tape locations were identified using accompanying User Guide. End tape location of **Record axis conditions (1-20)** are lower by 2 digits compared to the values in the User Guide. This was done for ease of manipulation: *Eg: I21 instead of I210, I211, .. , I219.* It was confirmed by opening the text file in NotePad 64 bit; does not violate dataset integrity.

```r
#Specifying a relative file path for the folder in working directory containing the mort data files
#This relative data path, has been specified in an attempt to ensure maximum reproducibility
```

```r
#on a different system, alleviating the need to manually set the working directory
#Just ensuring the MORTf folder to be inside the working directory, shall be sufficient
file_paths <- dir_ls("~/R/MORTf")
file_paths
```

```
## /home/rstudio/R/MORTf/Mort2018US.PubUse.txt
## /home/rstudio/R/MORTf/VS12MORT.DUSMCPUB
## /home/rstudio/R/MORTf/VS13MORT.DUSMCPUB
## /home/rstudio/R/MORTf/VS14MORT.DUSMCPUB
## /home/rstudio/R/MORTf/VS15MORT.DUSMCPUB
## /home/rstudio/R/MORTf/VS16MORT.DUSMCPUB
## /home/rstudio/R/MORTf/VS17MORT.DUSMCPUB
## /home/rstudio/R/MORTf/VS19MORT.DUSMCPUB_r20210304
```

```r
  VSMORT <- file_paths %>%
    map_df(function(path) {
      read_fwf(
        path,
        fwf_positions(
          start = c( 102, 20, 69, 79, 445, 488, 341, 344, 349, 354, 359, 364, 369, 374,
                     379, 384, 389, 394, 399, 404, 409, 414, 419, 424, 429, 434, 439),
          end = c( 105, 20, 69, 80, 446, 488, 342, 346, 351, 356, 361, 366, 371, 376,
                   381, 386, 391, 396, 401, 406, 411, 416, 433, 428, 429, 438, 443),
          col_names = c("Year",
                        "Resident_Status",
                        "Sex",
                        "Age_Recode_12",
                        "Race",
                        "Hispanic_Origin_Recode",
                        "Number_of_RA_conditions",
                        "RA1",
                        "RA2",
                        "RA3",
                        "RA4",
                        "RA5",
                        "RA6",
                        "RA7",
                        "RA8",
                        "RA9",
                        "RA10",
                        "RA11",
                        "RA12",
                        "RA13",
                        "RA14",
                        "RA15",
                        "RA16",
                        "RA17",
                        "RA18",
                        "RA19",
                        "RA20"
          )
        ),
        col_types = cols_only(
          Year= col_number(),
```

```
          Resident_Status = col_number(),
          Sex = col_factor(levels = c("M", "F")),
          Age_Recode_12 = col_number(),
          Race = col_number(),
          Hispanic_Origin_Recode = col_number(),
          Number_of_RA_conditions = col_number(),
          RA1 = col_character(),
          RA2 = col_character(),
          RA3 = col_character(),
          RA4 = col_character(),
          RA5 = col_character(),
          RA6 = col_character(),
          RA7 = col_character(),
          RA8 = col_character(),
          RA9 = col_character(),
          RA10 = col_character(),
          RA11 = col_character(),
          RA12 = col_character(),
          RA13 = col_character(),
          RA14 = col_character(),
          RA15 = col_character(),
          RA16 = col_character(),
          RA17 = col_character(),
          RA18 = col_character(),
          RA19 = col_character(),
          RA20 = col_character()
        )
      )
    })
  glimpse(VSMORT)
```

```
## Rows: 21,776,411
## Columns: 27
## $ Year                    <dbl> 2018, 2018, 2018, 2018, 2018, 2018, 2018, 2...
## $ Resident_Status         <dbl> 3, 1, 1, 1, 2, 2, 1, 1, 1, 1, 1, 1, 1, 1, 1...
## $ Sex                     <fct> F, M, M, M, F, F, M, M, M, F, F, F, F, F, F...
## $ Age_Recode_12           <dbl> 7, 5, 11, 11, 5, 5, 11, 11, 10, 11, 8, 8, 9...
## $ Race                    <dbl> 1, 3, 1, 1, 3, 1, 3, 1, 1, 1, 1, 1, 1, 1, 1...
## $ Hispanic_Origin_Recode  <dbl> 6, 8, 6, 6, 8, 6, 8, 6, 5, 6, 6, 6, 6, 6, 6...
## $ Number_of_RA_conditions <dbl> 3, 5, 1, 3, 8, 4, 1, 2, 1, 2, 1, 1, 2, 3, 7...
## $ RA1                     <chr> "A41", "V47", "C61", "A41", "K70", "V89", "...
## $ RA2                     <chr> "J18", "S02", NA, "I50", "D64", "S09", NA, ...
## $ RA3                     <chr> "J86", "S09", NA, "R68", "D68", "S19", NA, ...
## $ RA4                     <chr> NA, "T09", NA, NA, "E88", "T09", NA, NA, NA...
## $ RA5                     <chr> NA, "T14", NA, NA, "G93", NA, NA, NA, NA, N...
## $ RA6                     <chr> NA, NA, NA, NA, "K70", NA, NA, NA, NA, NA, ...
## $ RA7                     <chr> NA, NA, NA, NA, "R57", NA, NA, NA, NA, NA, ...
## $ RA8                     <chr> NA, NA, NA, NA, "R68", NA, NA, NA, NA, NA, ...
## $ RA9                     <chr> NA, NA, NA, NA, NA, NA, NA, NA, NA, NA, NA,...
## $ RA10                    <chr> NA, NA, NA, NA, NA, NA, NA, NA, NA, NA, NA,...
## $ RA11                    <chr> NA, NA, NA, NA, NA, NA, NA, NA, NA, NA, NA,...
## $ RA12                    <chr> NA, NA, NA, NA, NA, NA, NA, NA, NA, NA, NA,...
## $ RA13                    <chr> NA, NA, NA, NA, NA, NA, NA, NA, NA, NA, NA,...
## $ RA14                    <chr> NA, NA, NA, NA, NA, NA, NA, NA, NA, NA, NA,...
```

```
## $ RA15                       <chr> NA, NA, NA, NA, NA, NA, NA, NA, NA, NA, NA,...
## $ RA16                       <chr> NA, NA, NA, NA, NA, NA, NA, NA, NA, NA, NA,...
## $ RA17                       <chr> NA, NA, NA, NA, NA, NA, NA, NA, NA, NA, NA,...
## $ RA18                       <chr> NA, NA, NA, NA, NA, NA, NA, NA, NA, NA, NA,...
## $ RA19                       <chr> NA, NA, NA, NA, NA, NA, NA, NA, NA, NA, NA,...
## $ RA20                       <chr> NA, NA, NA, NA, NA, NA, NA, NA, NA, NA, NA,...
```

```
#Convert tibble into dataframe for ease of analysis
VSMORT <- as.data.frame(VSMORT)
str(VSMORT)
```

```
## 'data.frame':    21776411 obs. of  27 variables:
##  $ Year                    : num  2018 2018 2018 2018 2018 ...
##  $ Resident_Status         : num  3 1 1 1 2 2 1 1 1 1 ...
##  $ Sex                     : Factor w/ 2 levels "M","F": 2 1 1 1 2 2 1 1 1 2 ...
##  $ Age_Recode_12           : num  7 5 11 11 5 5 11 11 10 11 ...
##  $ Race                    : num  1 3 1 1 3 1 3 1 1 1 ...
##  $ Hispanic_Origin_Recode : num  6 8 6 6 8 6 8 6 5 6 ...
##  $ Number_of_RA_conditions: num  3 5 1 3 8 4 1 2 1 2 ...
##  $ RA1                     : chr  "A41" "V47" "C61" "A41" ...
##  $ RA2                     : chr  "J18" "S02" NA "I50" ...
##  $ RA3                     : chr  "J86" "S09" NA "R68" ...
##  $ RA4                     : chr  NA "T09" NA NA ...
##  $ RA5                     : chr  NA "T14" NA NA ...
##  $ RA6                     : chr  NA NA NA NA ...
##  $ RA7                     : chr  NA NA NA NA ...
##  $ RA8                     : chr  NA NA NA NA ...
##  $ RA9                     : chr  NA NA NA NA ...
##  $ RA10                    : chr  NA NA NA NA ...
##  $ RA11                    : chr  NA NA NA NA ...
##  $ RA12                    : chr  NA NA NA NA ...
##  $ RA13                    : chr  NA NA NA NA ...
##  $ RA14                    : chr  NA NA NA NA ...
##  $ RA15                    : chr  NA NA NA NA ...
##  $ RA16                    : chr  NA NA NA NA ...
##  $ RA17                    : chr  NA NA NA NA ...
##  $ RA18                    : chr  NA NA NA NA ...
##  $ RA19                    : chr  NA NA NA NA ...
##  $ RA20                    : chr  NA NA NA NA ...
##  - attr(*, "spec")=
##   .. cols_only(
##   ..   Year = col_number(),
##   ..   Resident_Status = col_number(),
##   ..   Sex = col_factor(levels = c("M", "F"), ordered = FALSE, include_na = FALSE),
##   ..   Age_Recode_12 = col_number(),
##   ..   Race = col_number(),
##   ..   Hispanic_Origin_Recode = col_number(),
##   ..   Number_of_RA_conditions = col_number(),
##   ..   RA1 = col_character(),
##   ..   RA2 = col_character(),
##   ..   RA3 = col_character(),
##   ..   RA4 = col_character(),
##   ..   RA5 = col_character(),
##   ..   RA6 = col_character(),
##   ..   RA7 = col_character(),
```

```
##   ..   RA8 = col_character(),
##   ..   RA9 = col_character(),
##   ..   RA10 = col_character(),
##   ..   RA11 = col_character(),
##   ..   RA12 = col_character(),
##   ..   RA13 = col_character(),
##   ..   RA14 = col_character(),
##   ..   RA15 = col_character(),
##   ..   RA16 = col_character(),
##   ..   RA17 = col_character(),
##   ..   RA18 = col_character(),
##   ..   RA19 = col_character(),
##   ..   RA20 = col_character()
##   .. )
```

Preprocessing

Tidy data Drop death records with one or more of the following: **Foreign Residents** *(-45,297)*, **Missing Age** *(-1,137)*, **Age of Death** $<$ **45** *(-1,526,209)*.

```
VSMORT_tidy <- VSMORT %>% filter(Resident_Status != 4, between(Age_Recode_12, 7, 11 ))
```

Make a new column "race_eth_nat" based on available data on Race and Hispanic-Origin; drop death records with **missing Race / Ethnicity / Nationality** *(-58,269)*.

```
#Create new column race_eth_nat
VSMORT_tidy <- VSMORT_tidy %>%
  mutate(race_eth_nat = case_when(Hispanic_Origin_Recode == 6 ~ "Non_Hispanic_White",
                              Hispanic_Origin_Recode == 7 ~ "Non_Hispanic_Black",
                              Race == 3 ~ "AmerIndian",
                              Race <= 2 & Hispanic_Origin_Recode <= 5 ~ "Hispanic",
                              Race == 4 ~ "Chinese",
                              Race == 5 ~ "Japanese",
                              Race == 7 ~ "Filipino",
                              Race == 18 ~ "Asian_Indian",
                              Race == 28 ~ "Korean",
                              Race == 48 ~ "Vietnamese",
                              Race == 6 | Race == 38 | Race == 58 | Race == 68
                              | Race == 78  ~ "OtherAPI" ))

#Drop rows with missing race_eth_nat (-58,269)
VSMORT_tidy <- VSMORT_tidy %>% filter(!is.na(race_eth_nat))
tail(VSMORT_tidy)
```

```
##          Year Resident_Status Sex Age_Recode_12 Race Hispanic_Origin_Recode
## 20145494 2019               1   F             9    1                      5
## 20145495 2019               1   M             9    1                      5
## 20145496 2019               1   M             8    1                      2
## 20145497 2019               2   M             7    2                      7
## 20145498 2019               1   F            10    2                      7
## 20145499 2019               2   F             7    2                      7
##          Number_of_RA_conditions RA1 RA2  RA3  RA4  RA5  RA6  RA7  RA8  RA9
## 20145494                       3 C34 C79  D64 <NA> <NA> <NA> <NA> <NA> <NA>
## 20145495                       4 I69 E78  I10  I46 <NA> <NA> <NA> <NA> <NA>
## 20145496                       2 C61 I11 <NA> <NA> <NA> <NA> <NA> <NA> <NA>
```

```
## 20145497                       5 D76 A41  A49  D64  N18 <NA> <NA> <NA> <NA>
## 20145498                       4 E85 I48  I50  N18 <NA> <NA> <NA> <NA> <NA>
## 20145499                       3 N18 D64  G12 <NA> <NA> <NA> <NA> <NA> <NA>
##          RA10 RA11 RA12 RA13 RA14 RA15 RA16 RA17 RA18 RA19 RA20
## 20145494 <NA> <NA> <NA> <NA> <NA> <NA> <NA> <NA> <NA> <NA> <NA>
## 20145495 <NA> <NA> <NA> <NA> <NA> <NA> <NA> <NA> <NA> <NA> <NA>
## 20145496 <NA> <NA> <NA> <NA> <NA> <NA> <NA> <NA> <NA> <NA> <NA>
## 20145497 <NA> <NA> <NA> <NA> <NA> <NA> <NA> <NA> <NA> <NA> <NA>
## 20145498 <NA> <NA> <NA> <NA> <NA> <NA> <NA> <NA> <NA> <NA> <NA>
## 20145499 <NA> <NA> <NA> <NA> <NA> <NA> <NA> <NA> <NA> <NA> <NA>
##                race_eth_nat
## 20145494           Hispanic
## 20145495           Hispanic
## 20145496           Hispanic
## 20145497 Non_Hispanic_Black
## 20145498 Non_Hispanic_Black
## 20145499 Non_Hispanic_Black
```

Find the population composition of Asian Indians and Not-Asian Indians

```r
#Collapse Non-Asian Indian categories into single category : Not_AI
  other = c("Non_Hispanic_White", "Non_Hispanic_Black", "AmerIndian", "Hispanic", "Chinese",
                      "Japanese", "Filipino", "Korean", "Vietnamese", "OtherAPI")
  VSMORT_tidy <- VSMORT_tidy %>%
    mutate(Asian_Indian_Status = fct_collapse(race_eth_nat,
                                              Not_Asian_Indian = other))

  #Tabulate Asian Indian status by frequency and percent population
  tab_AI_status <- VSMORT_tidy %>%
    count(Asian_Indian_Status) %>%
    mutate(Percent_population = n*100/sum(n))
  tab_AI_status
```

```
##   Asian_Indian_Status        n Percent_population
## 1    Not_Asian_Indian 20090038         99.7246978
## 2        Asian_Indian    55461          0.2753022
```

Further Analysis for **Multiple Causes of Death (MCOD)**: The maximum number of contributing causes of death *(Record Axis Conditions)* were found to be **15**. Hence, the remaining columns were dropped; as were those columns not required for analysis downstream.

```r
#Further for MCOD analysis
  #Find maximum number of record axis conditions
  Max_MCOD_count <- VSMORT_tidy %>%
  count(Number_of_RA_conditions) %>%
  arrange(desc(n))
  Max_MCOD_count
```

```
##    Number_of_RA_conditions       n
## 1                        1 4765823
## 2                        2 4729033
## 3                        3 4083281
## 4                        4 2897758
## 5                        5 1710459
## 6                        6  944066
## 7                        7  500879
## 8                        8  260235
```

```
## 9                          9  133235
## 10                        10   66015
## 11                        11   32111
## 12                        12   14969
## 13                        13    5751
## 14                        14    1778
## 15                        15      95
## 16                        16       7
## 17                        17       3
## 18                        19       1
```

```
#Remove columns with all NA
VSMORT_tidy <- VSMORT_tidy %>%  select_if(~!all(is.na(.)))
#Remove following columns; not needed for further analysis
VSMORT_tidy <- VSMORT_tidy %>%
  select(- Resident_Status, - Race, - Hispanic_Origin_Recode, - race_eth_nat)
str(VSMORT_tidy)
```

```
## 'data.frame':    20145499 obs. of  24 variables:
##  $ Year                    : num  2018 2018 2018 2018 2018 ...
##  $ Sex                     : Factor w/ 2 levels "M","F": 2 1 1 1 1 1 2 2 2 2 ...
##  $ Age_Recode_12           : num  7 11 11 11 11 10 11 8 8 9 ...
##  $ Number_of_RA_conditions: num  3 1 3 1 2 1 2 1 1 2 ...
##  $ RA1                     : chr  "A41" "C61" "A41" "A41" ...
##  $ RA2                     : chr  "J18" NA "I50" NA ...
##  $ RA3                     : chr  "J86" NA "R68" NA ...
##  $ RA4                     : chr  NA NA NA NA ...
##  $ RA5                     : chr  NA NA NA NA ...
##  $ RA6                     : chr  NA NA NA NA ...
##  $ RA7                     : chr  NA NA NA NA ...
##  $ RA8                     : chr  NA NA NA NA ...
##  $ RA9                     : chr  NA NA NA NA ...
##  $ RA10                    : chr  NA NA NA NA ...
##  $ RA11                    : chr  NA NA NA NA ...
##  $ RA12                    : chr  NA NA NA NA ...
##  $ RA13                    : chr  NA NA NA NA ...
##  $ RA14                    : chr  NA NA NA NA ...
##  $ RA15                    : chr  NA NA NA NA ...
##  $ RA16                    : chr  NA NA NA NA ...
##  $ RA17                    : chr  NA NA NA NA ...
##  $ RA18                    : chr  NA NA NA NA ...
##  $ RA19                    : chr  NA NA NA NA ...
##  $ Asian_Indian_Status     : Factor w/ 2 levels "Not_Asian_Indian",..: 1 1 1 1 1 1 1 1 1 1 ...
```

Any contributing **atherosclerotic-related disease** (either of **ischemic heart disease, ischemic stroke** or **atherosclerosis)** was identified using their ICD10 codes:- *I20 - I25, I63, and I70, respectively.* Similarly, any contributing causes by **diabetes mellitus** was identified by ICD codes *E10 - E14*.

Next, any mention of *atherosclerotic disease* was recoded as **"1"**, that of *diabetes mellitus* as **"2"**, and all *other contributing causes* as **"0"**

```
#Assign vector containing ICD codes to any atherosclerosis and diabetes mellitus
  #any_athero: any IHD (I20-I25), any ischemic stroke (I63), any atherosclerosis (I70)
  mcod_any_athero <- c ( "I20", "I21", "I22", "I23", "I24", "I25", "I63", "I70" )
  #any_diabetes_mellitus (E10-E14)
  mcod_any_dm<- c( "E10", "E11", "E12", "E13", "E14")
```

```r
#Recode any_athero with "1"; any_diabetes_mellitus with "2"; others as "0"
  VSMORT_ATH_DM <- VSMORT_tidy %>%
    mutate(across(paste0("RA", 1:15),
                  ~ case_when(. %in% mcod_any_dm ~ 2, . %in% mcod_any_athero ~ 1,
                              TRUE ~ 0)))
  tail(VSMORT_ATH_DM)
```

```
##          Year Sex Age_Recode_12 Number_of_RA_conditions RA1 RA2 RA3 RA4 RA5 RA6
## 20145494 2019   F             9                       3   0   0   0   0   0   0
## 20145495 2019   M             9                       4   0   0   0   0   0   0
## 20145496 2019   M             8                       2   0   0   0   0   0   0
## 20145497 2019   M             7                       5   0   0   0   0   0   0
## 20145498 2019   F            10                       4   0   0   0   0   0   0
## 20145499 2019   F             7                       3   0   0   0   0   0   0
##          RA7 RA8 RA9 RA10 RA11 RA12 RA13 RA14 RA15 RA16 RA17 RA18 RA19
## 20145494   0   0   0    0    0    0    0    0    0 <NA> <NA> <NA> <NA>
## 20145495   0   0   0    0    0    0    0    0    0 <NA> <NA> <NA> <NA>
## 20145496   0   0   0    0    0    0    0    0    0 <NA> <NA> <NA> <NA>
## 20145497   0   0   0    0    0    0    0    0    0 <NA> <NA> <NA> <NA>
## 20145498   0   0   0    0    0    0    0    0    0 <NA> <NA> <NA> <NA>
## 20145499   0   0   0    0    0    0    0    0    0 <NA> <NA> <NA> <NA>
##          Asian_Indian_Status
## 20145494    Not_Asian_Indian
## 20145495    Not_Asian_Indian
## 20145496    Not_Asian_Indian
## 20145497    Not_Asian_Indian
## 20145498    Not_Asian_Indian
## 20145499    Not_Asian_Indian
```

In the 2012-2019 dataset, there were **4,518,524** deaths **(22.43%)** due to atherosclerotic-related disease as contributing cause(s):

```r
#Analysing only for atherosclerosis:
  #Recode only for any_athero with "1"
  VSMORT_ATH <- VSMORT_tidy %>%
  mutate(across(paste0("RA", 1:15), ~ if_else(. %in% mcod_any_athero, 1, 0)))
  #Determine any atherosclerosis (ATH status)
  VSMORT_ATH <- VSMORT_ATH %>%
    mutate(ATH_status = case_when( RA1 == 1 | RA2 == 1 | RA3 == 1 | RA4 == 1 |
                                   RA5 == 1 | RA6 == 1 |RA7 == 1 |RA8 == 1 |
                                   RA9 == 1 |RA10 == 1 | RA11 == 1 | RA12 == 1 |
                                   RA13 == 1 |RA14 == 1 |RA15 == 1 ~ "ATH",
                                   TRUE ~ "Non-ATH"))
  #Table for ATH_status in dataset
  tab_ATH_count <- VSMORT_ATH %>%
    count(ATH_status) %>%
    mutate(Percent_population = n*100/sum(n))
  #Number of any_athero deaths is 4,518,524 (22.34%)
  tab_ATH_count
```

```
##   ATH_status        n Percent_population
## 1        ATH  4518524           22.42945
## 2    Non-ATH 15626975           77.57055
```

```r
#Further, based on Asian-Indian Status
tab_ATH_count_AI_vs_Not_AI <- VSMORT_ATH %>% count(Asian_Indian_Status, ATH_status)
tab_ATH_count_AI_vs_Not_AI
```

```
##   Asian_Indian_Status ATH_status        n
## 1    Not_Asian_Indian        ATH  4501961
## 2    Not_Asian_Indian    Non-ATH 15588077
## 3        Asian_Indian        ATH    16563
## 4        Asian_Indian    Non-ATH    38898
```

```r
#Table to calculate % deaths due to ATH among AI:
  tab_ATH_count_among_AI <- tab_ATH_count_AI_vs_Not_AI %>%
                            filter(Asian_Indian_Status == "Asian_Indian") %>%
                            mutate(Percent_population = n*100/sum(n))
  tab_ATH_count_among_AI
```

```
##   Asian_Indian_Status ATH_status     n Percent_population
## 1        Asian_Indian        ATH 16563           29.86423
## 2        Asian_Indian    Non-ATH 38898           70.13577
```

```r
  #Table to calculate % deaths due to ATH among NotAI:
  tab_ATH_count_among_NotAI <- tab_ATH_count_AI_vs_Not_AI %>%
                            filter(Asian_Indian_Status == "Not_Asian_Indian") %>%
                            mutate(Percent_population = n*100/sum(n))
  tab_ATH_count_among_NotAI
```

```
##   Asian_Indian_Status ATH_status        n Percent_population
## 1    Not_Asian_Indian        ATH  4501961           22.40892
## 2    Not_Asian_Indian    Non-ATH 15588077           77.59108
```

There were **2,017,221** deaths **(10.01%)** due to diabetes-mellitus as contributing cause(s):

```r
#Analysing only for diabetes mellitus:
  #Recode any diabetes with "2"
  VSMORT_DM <- VSMORT_tidy %>% mutate(across(paste0("RA", 1:15), ~ if_else(. %in% mcod_any_dm, 2, 0)))
  #Determine any diabetes (DM status)
  VSMORT_DM <- VSMORT_DM %>%
    mutate(DM_status = case_when( RA1 == 2 | RA2 == 2 | RA3 == 2 | RA4 == 2 |
                                  RA5 == 2 | RA6 == 2 |RA7 == 2 |RA8 == 2 |
                                  RA9 == 2 |RA10 == 2 | RA11 == 2 |RA12 == 2 |
                                  RA13 == 2 |RA14 == 2 |RA15 == 2 ~ "DM",
                                  TRUE ~ "Non-DM"))
  #Table for DM_status in dataset
  tab_DM_count <- VSMORT_DM %>%
    count(DM_status) %>%
    mutate(Percent_population = n*100/sum(n))
  #Number of any_diabetes deaths is 2,017,221 (10.01%)
  tab_DM_count
```

```
##   DM_status        n Percent_population
## 1        DM  2017221           10.01326
## 2    Non-DM 18128278           89.98674
```

```r
  #Further, based on Asian-Indian Status
  tab_DM_count_AI_vs_Not_AI <- VSMORT_DM %>%
    count(Asian_Indian_Status, DM_status)
  tab_DM_count_AI_vs_Not_AI
```

```
##   Asian_Indian_Status DM_status        n
## 1    Not_Asian_Indian        DM  2008113
## 2    Not_Asian_Indian    Non-DM 18081925
## 3        Asian_Indian        DM     9108
## 4        Asian_Indian    Non-DM    46353
```

```
#Table to calculate % deaths due to DM among AI:
   tab_DM_count_among_AI <- tab_DM_count_AI_vs_Not_AI %>%
                              filter(Asian_Indian_Status == "Asian_Indian") %>%
                              mutate(Percent_population = n*100/sum(n))
   tab_DM_count_among_AI
```

```
##   Asian_Indian_Status DM_status     n Percent_population
## 1        Asian_Indian        DM  9108           16.42235
## 2        Asian_Indian    Non-DM 46353           83.57765
```

```
   #Table to calculate % deaths due to DM among NotAI:
   tab_DM_count_among_NotAI <- tab_DM_count_AI_vs_Not_AI %>%
                              filter(Asian_Indian_Status == "Not_Asian_Indian") %>%
                              mutate(Percent_population = n*100/sum(n))
   tab_DM_count_among_NotAI
```

```
##   Asian_Indian_Status DM_status        n Percent_population
## 1    Not_Asian_Indian        DM  2008113           9.995566
## 2    Not_Asian_Indian    Non-DM 18081925          90.004434
```

In the 2012-2019 dataset, the number of deaths due to *atherosclerotic-disease(s)* where *diabetes-mellitus* also contributed was **830,865 (18.39%)**.

```
#Join DM_status column and ATH_status column to the main data.frame VS17MORT_ATH_DM:
#Used column bind function in dplyr to match columns by position in each data.frame :
        #valid as row positions are same in each df
#Although using the inner_join() would have been a better choice, it could not
        #be done due to large size of each data.frame
Only_DM_col <- VSMORT_DM %>% select(DM_status)
Only_ATH_col <- VSMORT_ATH %>% select(ATH_status)
VSMORT_ATH_DM <- bind_cols(VSMORT_ATH_DM, Only_DM_col)
VSMORT_ATH_DM <- bind_cols(VSMORT_ATH_DM, Only_ATH_col)
tail(VSMORT_ATH_DM)
```

```
##          Year Sex Age_Recode_12 Number_of_RA_conditions RA1 RA2 RA3 RA4 RA5 RA6
## 20145494 2019   F             9                       3   0   0   0   0   0   0
## 20145495 2019   M             9                       4   0   0   0   0   0   0
## 20145496 2019   M             8                       2   0   0   0   0   0   0
## 20145497 2019   M             7                       5   0   0   0   0   0   0
## 20145498 2019   F            10                       4   0   0   0   0   0   0
## 20145499 2019   F             7                       3   0   0   0   0   0   0
##          RA7 RA8 RA9 RA10 RA11 RA12 RA13 RA14 RA15 RA16 RA17 RA18 RA19
## 20145494   0   0   0    0    0    0    0    0    0 <NA> <NA> <NA> <NA>
## 20145495   0   0   0    0    0    0    0    0    0 <NA> <NA> <NA> <NA>
## 20145496   0   0   0    0    0    0    0    0    0 <NA> <NA> <NA> <NA>
## 20145497   0   0   0    0    0    0    0    0    0 <NA> <NA> <NA> <NA>
## 20145498   0   0   0    0    0    0    0    0    0 <NA> <NA> <NA> <NA>
## 20145499   0   0   0    0    0    0    0    0    0 <NA> <NA> <NA> <NA>
##          Asian_Indian_Status DM_status ATH_status
## 20145494    Not_Asian_Indian    Non-DM    Non-ATH
## 20145495    Not_Asian_Indian    Non-DM    Non-ATH
```

```
## 20145496    Not_Asian_Indian    Non-DM    Non-ATH
## 20145497    Not_Asian_Indian    Non-DM    Non-ATH
## 20145498    Not_Asian_Indian    Non-DM    Non-ATH
## 20145499    Not_Asian_Indian    Non-DM    Non-ATH
```

```
#To find number of DM-related deaths among ATH-related deaths in entire df
tab_count_DM_in_ATH_by_AI_status <- VSMORT_ATH_DM %>%
                                    count(ATH_status, DM_status, Asian_Indian_Status) %>%
                 filter(ATH_status == "ATH") %>% mutate(Percent_population = n*100/sum(n))
#Number of DM deaths among ATH deaths is 830,865 (18.39%)
tab_count_DM_in_ATH_by_AI_status
```

```
##   ATH_status DM_status Asian_Indian_Status       n Percent_population
## 1        ATH        DM    Not_Asian_Indian  826262         18.2861040
## 2        ATH        DM        Asian_Indian    4603          0.1018695
## 3        ATH    Non-DM    Not_Asian_Indian 3675699         81.3473382
## 4        ATH    Non-DM        Asian_Indian   11960          0.2646882
```

```
   #To find %ATH&DM in AI:
   tab_count_ATH_DM_AI <- tab_count_DM_in_ATH_by_AI_status %>%
     filter(Asian_Indian_Status == "Asian_Indian") %>%  select( -Percent_population) %>%
                              mutate(Percent_population = n*100/sum(n))
   tab_count_ATH_DM_AI
```

```
##   ATH_status DM_status Asian_Indian_Status     n Percent_population
## 1        ATH        DM        Asian_Indian  4603           27.79086
## 2        ATH    Non-DM        Asian_Indian 11960           72.20914
```

```
   #To find %ATH&DM in Not-AI:
   tab_count_ATH_DM_NotAI <- tab_count_DM_in_ATH_by_AI_status %>%
     filter(Asian_Indian_Status == "Not_Asian_Indian") %>%  select( -Percent_population) %>%
                              mutate(Percent_population = n*100/sum(n))
   tab_count_ATH_DM_NotAI
```

```
##   ATH_status DM_status Asian_Indian_Status       n Percent_population
## 1        ATH        DM    Not_Asian_Indian  826262           18.35338
## 2        ATH    Non-DM    Not_Asian_Indian 3675699           81.64662
```

Split dataset based on Asian Indian status for further analysis (calculation of Rho, p-values, % excess diabetes, graph plot):

```
#Create dataframe containing only Asian Indians
  AI <- VSMORT_ATH_DM %>% filter(Asian_Indian_Status == "Asian_Indian")
  glimpse(AI)
```

```
## Rows: 55,461
## Columns: 26
## $ Year                      <dbl> 2018, 2018, 2018, 2018, 2018, 2018, 2018, 2...
## $ Sex                       <fct> M, M, M, M, M, M, M, M, F, F, M, M, M, M, M...
## $ Age_Recode_12             <dbl> 9, 9, 8, 10, 11, 11, 10, 8, 11, 7, 8, 8, 10...
## $ Number_of_RA_conditions <dbl> 6, 2, 5, 2, 1, 3, 1, 1, 2, 4, 1, 1, 7, 4, 2...
## $ RA1                       <dbl> 1, 0, 0, 0, 0, 1, 0, 0, 0, 0, 0, 0, 0, 0, 0...
## $ RA2                       <dbl> 0, 0, 0, 0, 0, 0, 0, 0, 0, 0, 0, 0, 0, 0, 0...
## $ RA3                       <dbl> 0, 0, 0, 0, 0, 0, 0, 0, 0, 0, 0, 0, 0, 0, 0...
## $ RA4                       <dbl> 0, 0, 0, 0, 0, 0, 0, 0, 0, 0, 0, 0, 0, 0, 0...
## $ RA5                       <dbl> 0, 0, 0, 0, 0, 0, 0, 0, 0, 0, 0, 0, 0, 0, 0...
## $ RA6                       <dbl> 0, 0, 0, 0, 0, 0, 0, 0, 0, 0, 0, 0, 0, 0, 0...
## $ RA7                       <dbl> 0, 0, 0, 0, 0, 0, 0, 0, 0, 0, 0, 0, 0, 0, 0...
```

```
## $ RA8                       <dbl> 0, 0, 0, 0, 0, 0, 0, 0, 0, 0, 0, 0, 0, 0, 0...
## $ RA9                       <dbl> 0, 0, 0, 0, 0, 0, 0, 0, 0, 0, 0, 0, 0, 0, 0...
## $ RA10                      <dbl> 0, 0, 0, 0, 0, 0, 0, 0, 0, 0, 0, 0, 0, 0, 0...
## $ RA11                      <dbl> 0, 0, 0, 0, 0, 0, 0, 0, 0, 0, 0, 0, 0, 0, 0...
## $ RA12                      <dbl> 0, 0, 0, 0, 0, 0, 0, 0, 0, 0, 0, 0, 0, 0, 0...
## $ RA13                      <dbl> 0, 0, 0, 0, 0, 0, 0, 0, 0, 0, 0, 0, 0, 0, 0...
## $ RA14                      <dbl> 0, 0, 0, 0, 0, 0, 0, 0, 0, 0, 0, 0, 0, 0, 0...
## $ RA15                      <dbl> 0, 0, 0, 0, 0, 0, 0, 0, 0, 0, 0, 0, 0, 0, 0...
## $ RA16                      <chr> NA, NA, NA, NA, NA, NA, NA, NA, NA, NA, NA,...
## $ RA17                      <chr> NA, NA, NA, NA, NA, NA, NA, NA, NA, NA, NA,...
## $ RA18                      <chr> NA, NA, NA, NA, NA, NA, NA, NA, NA, NA, NA,...
## $ RA19                      <chr> NA, NA, NA, NA, NA, NA, NA, NA, NA, NA, NA,...
## $ Asian_Indian_Status       <fct> Asian_Indian, Asian_Indian, Asian_Indian, A...
## $ DM_status                 <chr> "Non-DM", "Non-DM", "Non-DM", "Non-DM", "No...
## $ ATH_status                <chr> "ATH", "Non-ATH", "Non-ATH", "Non-ATH", "No...
```

```
#Create dataframe containing only Not-Asian Indians
  Not_AI <- VSMORT_ATH_DM %>% filter(Asian_Indian_Status == "Not_Asian_Indian")
  glimpse(Not_AI)
```

```
## Rows: 20,090,038
## Columns: 26
## $ Year                      <dbl> 2018, 2018, 2018, 2018, 2018, 2018, 2018, 2...
## $ Sex                       <fct> F, M, M, M, M, M, F, F, F, F, F, F, F, F, F...
## $ Age_Recode_12             <dbl> 7, 11, 11, 11, 11, 10, 11, 8, 8, 9, 11, 11,...
## $ Number_of_RA_conditions   <dbl> 3, 1, 3, 1, 2, 1, 2, 1, 1, 2, 7, 3, 2, 3, 6...
## $ RA1                       <dbl> 0, 0, 0, 0, 2, 0, 1, 0, 0, 0, 0, 1, 0, 0, 0...
## $ RA2                       <dbl> 0, 0, 0, 0, 0, 0, 0, 0, 0, 0, 0, 0, 0, 0, 1...
## $ RA3                       <dbl> 0, 0, 0, 0, 0, 0, 0, 0, 0, 0, 1, 0, 0, 0, 0...
## $ RA4                       <dbl> 0, 0, 0, 0, 0, 0, 0, 0, 0, 0, 0, 0, 0, 0, 0...
## $ RA5                       <dbl> 0, 0, 0, 0, 0, 0, 0, 0, 0, 0, 0, 0, 0, 0, 0...
## $ RA6                       <dbl> 0, 0, 0, 0, 0, 0, 0, 0, 0, 0, 0, 0, 0, 0, 0...
## $ RA7                       <dbl> 0, 0, 0, 0, 0, 0, 0, 0, 0, 0, 0, 0, 0, 0, 0...
## $ RA8                       <dbl> 0, 0, 0, 0, 0, 0, 0, 0, 0, 0, 0, 0, 0, 0, 0...
## $ RA9                       <dbl> 0, 0, 0, 0, 0, 0, 0, 0, 0, 0, 0, 0, 0, 0, 0...
## $ RA10                      <dbl> 0, 0, 0, 0, 0, 0, 0, 0, 0, 0, 0, 0, 0, 0, 0...
## $ RA11                      <dbl> 0, 0, 0, 0, 0, 0, 0, 0, 0, 0, 0, 0, 0, 0, 0...
## $ RA12                      <dbl> 0, 0, 0, 0, 0, 0, 0, 0, 0, 0, 0, 0, 0, 0, 0...
## $ RA13                      <dbl> 0, 0, 0, 0, 0, 0, 0, 0, 0, 0, 0, 0, 0, 0, 0...
## $ RA14                      <dbl> 0, 0, 0, 0, 0, 0, 0, 0, 0, 0, 0, 0, 0, 0, 0...
## $ RA15                      <dbl> 0, 0, 0, 0, 0, 0, 0, 0, 0, 0, 0, 0, 0, 0, 0...
## $ RA16                      <chr> NA, NA, NA, NA, NA, NA, NA, NA, NA, NA, NA,...
## $ RA17                      <chr> NA, NA, NA, NA, NA, NA, NA, NA, NA, NA, NA,...
## $ RA18                      <chr> NA, NA, NA, NA, NA, NA, NA, NA, NA, NA, NA,...
## $ RA19                      <chr> NA, NA, NA, NA, NA, NA, NA, NA, NA, NA, NA,...
## $ Asian_Indian_Status       <fct> Not_Asian_Indian, Not_Asian_Indian, Not_Asi...
## $ DM_status                 <chr> "Non-DM", "Non-DM", "Non-DM", "Non-DM", "DM...
## $ ATH_status                <chr> "Non-ATH", "Non-ATH", "Non-ATH", "Non-ATH",...
```

Tetrachoric correlation (Rho): How strongly do contributing causes of atheroslerotic-disease(s) and diabetes mellitus cluster based on Asian Indian status? For **Asian Indians**:

```
 #Calculate tetrachoric correlation for ATH and DM in AI
  Rho_AI <- polychor(AI$ATH_status, AI$DM_status, ML = TRUE, std.err = TRUE)
  Rho_AI
```

```
##
## Polychoric Correlation, ML est. = 0.3597 (0.0074)
##
##   Row Threshold
##   Threshold Std.Err.
##     -0.5283 0.005601
##
##
##   Column Threshold
##   Threshold Std.Err.
##     -0.9772 0.006357
```

```r
#Find p-value for cluster in AI
tab_contingency_AI <-  table(AI$ATH_status, AI$DM_status)
tab_contingency_AI
```

```
##
##              DM Non-DM
##   ATH      4603  11960
##   Non-ATH  4505  34393
```

```r
p_val_AI <- fisher.test(tab_contingency_AI)
p_val_AI
```

```
##
##  Fisher's Exact Test for Count Data
##
## data:  tab_contingency_AI
## p-value < 2.2e-16
## alternative hypothesis: true odds ratio is not equal to 1
## 95 percent confidence interval:
##  2.805224 3.077403
## sample estimates:
## odds ratio
##    2.93823
```

For **Not Asian Indians**:

```r
#Calculate tetrachoric correlation for ATH and DM in Not-AI
Rho_Not_AI <- polychor(Not_AI$ATH_status, Not_AI$DM_status, ML = TRUE, std.err = TRUE)
Rho_Not_AI
```

```
##
## Polychoric Correlation, ML est. = 0.3101 (0.0004703)
##
##   Row Threshold
##   Threshold  Std.Err.
##     -0.7585 0.0003109
##
##
##   Column Threshold
##   Threshold  Std.Err.
##      -1.282 0.0003814
```

```r
#Find p-value for cluster in Not-AI
tab_contingency_Not_AI <-  table(Not_AI$ATH_status, Not_AI$DM_status)
```

```r
tab_contingency_Not_AI
```

```
##
##                  DM   Non-DM
##   ATH       826262  3675699
##   Non-ATH  1181851 14406226
```

```r
p_val_Not_AI <- fisher.test(tab_contingency_Not_AI)
p_val_Not_AI
```

```
##
##  Fisher's Exact Test for Count Data
##
## data:  tab_contingency_Not_AI
## p-value < 2.2e-16
## alternative hypothesis: true odds ratio is not equal to 1
## 95 percent confidence interval:
##  2.731669 2.748232
## sample estimates:
## odds ratio
##   2.740087
```

Generating the table for publication:

```r
#To create table for publication:
table_for_pub <- VSMORT_ATH_DM %>%
 mutate(Cluster = case_when(ATH_status == "ATH" & DM_status =="DM" ~ "ATH&DM",
                            ATH_status == "ATH" & DM_status =="Non-DM" ~ "ATH&Non-DM",
                            ATH_status == "Non-ATH" & DM_status =="DM" ~ "Non-ATH&DM",
                            ATH_status == "Non-ATH" & DM_status =="Non-DM" ~ "Non-ATH&Non-DM"))
glimpse(table_for_pub)
```

```
## Rows: 20,145,499
## Columns: 27
## $ Year                     <dbl> 2018, 2018, 2018, 2018, 2018, 2018, 2018, 2...
## $ Sex                      <fct> F, M, M, M, M, M, F, F, F, F, F, F, F, F, F...
## $ Age_Recode_12            <dbl> 7, 11, 11, 11, 11, 10, 11, 8, 8, 9, 11, 11,...
## $ Number_of_RA_conditions <dbl> 3, 1, 3, 1, 2, 1, 2, 1, 1, 2, 7, 3, 2, 3, 6...
## $ RA1                      <dbl> 0, 0, 0, 0, 2, 0, 1, 0, 0, 0, 0, 1, 0, 0, 0...
## $ RA2                      <dbl> 0, 0, 0, 0, 0, 0, 0, 0, 0, 0, 0, 0, 0, 0, 1...
## $ RA3                      <dbl> 0, 0, 0, 0, 0, 0, 0, 0, 0, 0, 1, 0, 0, 0, 0...
## $ RA4                      <dbl> 0, 0, 0, 0, 0, 0, 0, 0, 0, 0, 0, 0, 0, 0, 0...
## $ RA5                      <dbl> 0, 0, 0, 0, 0, 0, 0, 0, 0, 0, 0, 0, 0, 0, 0...
## $ RA6                      <dbl> 0, 0, 0, 0, 0, 0, 0, 0, 0, 0, 0, 0, 0, 0, 0...
## $ RA7                      <dbl> 0, 0, 0, 0, 0, 0, 0, 0, 0, 0, 0, 0, 0, 0, 0...
## $ RA8                      <dbl> 0, 0, 0, 0, 0, 0, 0, 0, 0, 0, 0, 0, 0, 0, 0...
## $ RA9                      <dbl> 0, 0, 0, 0, 0, 0, 0, 0, 0, 0, 0, 0, 0, 0, 0...
## $ RA10                     <dbl> 0, 0, 0, 0, 0, 0, 0, 0, 0, 0, 0, 0, 0, 0, 0...
## $ RA11                     <dbl> 0, 0, 0, 0, 0, 0, 0, 0, 0, 0, 0, 0, 0, 0, 0...
## $ RA12                     <dbl> 0, 0, 0, 0, 0, 0, 0, 0, 0, 0, 0, 0, 0, 0, 0...
## $ RA13                     <dbl> 0, 0, 0, 0, 0, 0, 0, 0, 0, 0, 0, 0, 0, 0, 0...
## $ RA14                     <dbl> 0, 0, 0, 0, 0, 0, 0, 0, 0, 0, 0, 0, 0, 0, 0...
## $ RA15                     <dbl> 0, 0, 0, 0, 0, 0, 0, 0, 0, 0, 0, 0, 0, 0, 0...
## $ RA16                     <chr> NA, NA, NA, NA, NA, NA, NA, NA, NA, NA, NA,...
## $ RA17                     <chr> NA, NA, NA, NA, NA, NA, NA, NA, NA, NA, NA,...
## $ RA18                     <chr> NA, NA, NA, NA, NA, NA, NA, NA, NA, NA, NA,...
```

```
## $ RA19                    <chr> NA, NA, NA, NA, NA, NA, NA, NA, NA, NA, NA,...
## $ Asian_Indian_Status     <fct> Not_Asian_Indian, Not_Asian_Indian, Not_Asi...
## $ DM_status               <chr> "Non-DM", "Non-DM", "Non-DM", "Non-DM", "DM...
## $ ATH_status              <chr> "Non-ATH", "Non-ATH", "Non-ATH", "Non-ATH",...
## $ Cluster                 <chr> "Non-ATH&Non-DM", "Non-ATH&Non-DM", "Non-AT...
```

```
table_cluster_AI <- table_for_pub %>%
                    filter (Asian_Indian_Status == "Asian_Indian") %>% count(Cluster) %>%
                    mutate(Percent_population = n*100/sum(n))
table_cluster_AI
```

```
##          Cluster     n Percent_population
## 1          ATH&DM  4603           8.299526
## 2      ATH&Non-DM 11960          21.564703
## 3      Non-ATH&DM  4505           8.122825
## 4 Non-ATH&Non-DM 34393          62.012946
```

```
table_cluster_NotAI <- table_for_pub %>%
                    filter (Asian_Indian_Status == "Not_Asian_Indian") %>% count(Cluster) %>%
                    mutate(Percent_population = n*100/sum(n))
table_cluster_NotAI
```

```
##          Cluster        n Percent_population
## 1          ATH&DM   826262           4.112795
## 2      ATH&Non-DM  3675699          18.296128
## 3      Non-ATH&DM  1181851           5.882771
## 4 Non-ATH&Non-DM 14406226          71.708306
```

p-value for **difference** in cluster between Asian Indians and Not-Asian Indians:

```
#Find p-value for cluster between AI and Not-AI
#Create cluster column
VSMORT_ATH_DM <- VSMORT_ATH_DM %>%
mutate(Cluster = case_when(ATH_status == "ATH" & DM_status =="DM" ~ 3, TRUE ~ 4 ))
tail(VSMORT_ATH_DM)
```

```
##          Year Sex Age_Recode_12 Number_of_RA_conditions RA1 RA2 RA3 RA4 RA5 RA6
## 20145494 2019   F             9                       3   0   0   0   0   0   0
## 20145495 2019   M             9                       4   0   0   0   0   0   0
## 20145496 2019   M             8                       2   0   0   0   0   0   0
## 20145497 2019   M             7                       5   0   0   0   0   0   0
## 20145498 2019   F            10                       4   0   0   0   0   0   0
## 20145499 2019   F             7                       3   0   0   0   0   0   0
##          RA7 RA8 RA9 RA10 RA11 RA12 RA13 RA14 RA15 RA16 RA17 RA18 RA19
## 20145494   0   0   0    0    0    0    0    0    0 <NA> <NA> <NA> <NA>
## 20145495   0   0   0    0    0    0    0    0    0 <NA> <NA> <NA> <NA>
## 20145496   0   0   0    0    0    0    0    0    0 <NA> <NA> <NA> <NA>
## 20145497   0   0   0    0    0    0    0    0    0 <NA> <NA> <NA> <NA>
## 20145498   0   0   0    0    0    0    0    0    0 <NA> <NA> <NA> <NA>
## 20145499   0   0   0    0    0    0    0    0    0 <NA> <NA> <NA> <NA>
##          Asian_Indian_Status DM_status ATH_status Cluster
## 20145494    Not_Asian_Indian    Non-DM    Non-ATH       4
## 20145495    Not_Asian_Indian    Non-DM    Non-ATH       4
## 20145496    Not_Asian_Indian    Non-DM    Non-ATH       4
## 20145497    Not_Asian_Indian    Non-DM    Non-ATH       4
## 20145498    Not_Asian_Indian    Non-DM    Non-ATH       4
## 20145499    Not_Asian_Indian    Non-DM    Non-ATH       4
```

```
VSMORT_ATH_DM$Asian_Indian_Status <- fct_relevel(VSMORT_ATH_DM$Asian_Indian_Status,
                                                 c("Asian_Indian", "Not_Asian_Indian"))
#p-value (2.2e-16) (odds ratio 2.193656)
tab_contingency_AI_vs_Not_AI<-  table(VSMORT_ATH_DM$Asian_Indian_Status, VSMORT_ATH_DM$Cluster)
tab_contingency_AI_vs_Not_AI
```

```
##
##                          3        4
##   Asian_Indian        4603    50858
##   Not_Asian_Indian   826262 19263776
```

```
p_val_AI_vs_Not_AI <- fisher.test(tab_contingency_AI_vs_Not_AI)
p_val_AI_vs_Not_AI
```

```
##
##  Fisher's Exact Test for Count Data
##
## data:  tab_contingency_AI_vs_Not_AI
## p-value < 2.2e-16
## alternative hypothesis: true odds ratio is not equal to 1
## 95 percent confidence interval:
##  2.046921 2.174981
## sample estimates:
## odds ratio
##   2.110247
```

% Excess deaths due to diabetes in atherosclerotic disease-related *vs.* unrelated deaths Further analysis to rule out any spurious association: (i) Find the % excess deaths due to diabetes mellitus when atherosclerotic-disease(s) also contributes, stratified by age group and sex; (ii) Find the standard error in % difference using the formula: sqrt[DM&ATH_fract * (1 - DM&ATH_fract) / Total_ATH_deaths + DM&NonATH_fract * (1 - DM&NonATH_fract) / Total_NonATH_deaths]

```
#1. Find total deaths due to ATH and ATH-Unrelated based on Asian Indian status
total_col <- VSMORT_ATH_DM %>%
count(Asian_Indian_Status, Age_Recode_12, Sex, ATH_status) %>%
select(n)
#2. Find total deaths due to (ATH + DM) and (ATH-Unrelated + DM) based on Asian Indian status
tab0<- VSMORT_ATH_DM %>%
  count(Asian_Indian_Status, Age_Recode_12, Sex, ATH_status, DM_status) %>%
  filter(DM_status == "DM")
#3. Bind total deaths column to tab1 based on position, find percentage of
    #deaths where DM also contributes
tab0 <- bind_cols(tab0, total_col) %>%
  rename( "Frequency" = "n...6", "Total" = "n...7") %>%
  mutate(Percent_pop = Frequency*100/Total)
tab0
```

```
##    Asian_Indian_Status Age_Recode_12 Sex ATH_status DM_status Frequency   Total
## 1         Asian_Indian             7   M        ATH        DM       185     800
## 2         Asian_Indian             7   M    Non-ATH        DM       186    2077
## 3         Asian_Indian             7   F        ATH        DM        42     117
## 4         Asian_Indian             7   F    Non-ATH        DM        86    1226
## 5         Asian_Indian             8   M        ATH        DM       567    1796
## 6         Asian_Indian             8   M    Non-ATH        DM       460    3606
## 7         Asian_Indian             8   F        ATH        DM       175     473
```

```
## 8         Asian_Indian                8   F    Non-ATH         DM        209    2176
## 9         Asian_Indian                9   M        ATH         DM        936    2812
## 10        Asian_Indian                9   M    Non-ATH         DM        651    5226
## 11        Asian_Indian                9   F        ATH         DM        371    1098
## 12        Asian_Indian                9   F    Non-ATH         DM        477    3795
## 13        Asian_Indian               10   M        ATH         DM        910    3333
## 14        Asian_Indian               10   M    Non-ATH         DM        831    6332
## 15        Asian_Indian               10   F        ATH         DM        609    2070
## 16        Asian_Indian               10   F    Non-ATH         DM        742    5429
## 17        Asian_Indian               11   M        ATH         DM        433    2055
## 18        Asian_Indian               11   M    Non-ATH         DM        371    4012
## 19        Asian_Indian               11   F        ATH         DM        375    2009
## 20        Asian_Indian               11   F    Non-ATH         DM        492    5019
## 21    Not_Asian_Indian                7   M        ATH         DM      31298  162254
## 22    Not_Asian_Indian                7   M    Non-ATH         DM      47362  668620
## 23    Not_Asian_Indian                7   F        ATH         DM      14974   62619
## 24    Not_Asian_Indian                7   F    Non-ATH         DM      32377  472850
## 25    Not_Asian_Indian                8   M        ATH         DM      88178  404792
## 26    Not_Asian_Indian                8   M    Non-ATH         DM     108990 1321438
## 27    Not_Asian_Indian                8   F        ATH         DM      43588  167672
## 28    Not_Asian_Indian                8   F    Non-ATH         DM      79029  947476
## 29    Not_Asian_Indian                9   M        ATH         DM     141083  581588
## 30    Not_Asian_Indian                9   M    Non-ATH         DM     155445 1684326
## 31    Not_Asian_Indian                9   F        ATH         DM      75301  300274
## 32    Not_Asian_Indian                9   F    Non-ATH         DM     126824 1402775
## 33    Not_Asian_Indian               10   M        ATH         DM     142785  692364
## 34    Not_Asian_Indian               10   M    Non-ATH         DM     160073 1920996
## 35    Not_Asian_Indian               10   F        ATH         DM      95688  494749
## 36    Not_Asian_Indian               10   F    Non-ATH         DM     165945 2027871
## 37    Not_Asian_Indian               11   M        ATH         DM      94296  711185
## 38    Not_Asian_Indian               11   M    Non-ATH         DM     117073 1840651
## 39    Not_Asian_Indian               11   F        ATH         DM      99071  924464
## 40    Not_Asian_Indian               11   F    Non-ATH         DM     188733 3301074
##    Percent_pop
## 1    23.125000
## 2     8.955224
## 3    35.897436
## 4     7.014682
## 5    31.570156
## 6    12.756517
## 7    36.997886
## 8     9.604779
## 9    33.285917
## 10   12.456946
## 11   33.788707
## 12   12.569170
## 13   27.302730
## 14   13.123816
## 15   29.420290
## 16   13.667342
## 17   21.070560
## 18    9.247258
## 19   18.666003
## 20    9.802750
```

```
## 21   19.289509
## 22    7.083545
## 23   23.912870
## 24    6.847203
## 25   21.783533
## 26    8.247833
## 27   25.995992
## 28    8.341003
## 29   24.258238
## 30    9.228914
## 31   25.077429
## 32    9.040937
## 33   20.622823
## 34    8.332813
## 35   19.340716
## 36    8.183213
## 37   13.258997
## 38    6.360413
## 39   10.716588
## 40    5.717321
```

```r
#4. Find excess % deaths due to DM in ATH-related deaths; use tab1 later to plot graph
tab1 <- tab0 %>%
  #Create a lead column for data transformation
  mutate(Lead_percent_pop = lead(Percent_pop)) %>%
  #Filter only the odd numbered rows
  filter(row_number() %% 2 == 1) %>%
  #Find excess%
  mutate(Excess_percent_deaths = Percent_pop - Lead_percent_pop) %>%
  #Include only columns relevant for plotting graph
  select(-ATH_status, -DM_status, -Frequency, -Total)
tab1
```

```
##    Asian_Indian_Status Age_Recode_12 Sex Percent_pop Lead_percent_pop
## 1         Asian_Indian             7   M    23.12500         8.955224
## 2         Asian_Indian             7   F    35.89744         7.014682
## 3         Asian_Indian             8   M    31.57016        12.756517
## 4         Asian_Indian             8   F    36.99789         9.604779
## 5         Asian_Indian             9   M    33.28592        12.456946
## 6         Asian_Indian             9   F    33.78871        12.569170
## 7         Asian_Indian            10   M    27.30273        13.123816
## 8         Asian_Indian            10   F    29.42029        13.667342
## 9         Asian_Indian            11   M    21.07056         9.247258
## 10        Asian_Indian            11   F    18.66600         9.802750
## 11    Not_Asian_Indian             7   M    19.28951         7.083545
## 12    Not_Asian_Indian             7   F    23.91287         6.847203
## 13    Not_Asian_Indian             8   M    21.78353         8.247833
## 14    Not_Asian_Indian             8   F    25.99599         8.341003
## 15    Not_Asian_Indian             9   M    24.25824         9.228914
## 16    Not_Asian_Indian             9   F    25.07743         9.040937
## 17    Not_Asian_Indian            10   M    20.62282         8.332813
## 18    Not_Asian_Indian            10   F    19.34072         8.183213
## 19    Not_Asian_Indian            11   M    13.25900         6.360413
## 20    Not_Asian_Indian            11   F    10.71659         5.717321
##    Excess_percent_deaths
```

```
## 1               14.169776
## 2               28.882754
## 3               18.813639
## 4               27.393106
## 5               20.828971
## 6               21.219537
## 7               14.178915
## 8               15.752948
## 9               11.823301
## 10               8.863253
## 11              12.205964
## 12              17.065667
## 13              13.535700
## 14              17.654989
## 15              15.029324
## 16              16.036493
## 17              12.290010
## 18              11.157503
## 19               6.898585
## 20               4.999267
```

```
#5. Find Standard Error (SE)
SE <- tab0 %>%
  #Select only relevant columns
  select(-DM_status, -ATH_status)%>%
  #For SE calculation:
  mutate(se_calc_1 = Percent_pop*(100 - Percent_pop)/Total) %>%
  #Create a lead column for data transformation
  mutate(se_calc_2 = lead(se_calc_1)) %>%
  #Filter only the odd numbered rows
  filter(row_number() %% 2 == 1) %>%
  mutate(SE = sqrt(se_calc_1 + se_calc_2))
SE
```

```
##    Asian_Indian_Status Age_Recode_12 Sex Frequency  Total Percent_pop
## 1         Asian_Indian             7   M       185    800    23.12500
## 2         Asian_Indian             7   F        42    117    35.89744
## 3         Asian_Indian             8   M       567   1796    31.57016
## 4         Asian_Indian             8   F       175    473    36.99789
## 5         Asian_Indian             9   M       936   2812    33.28592
## 6         Asian_Indian             9   F       371   1098    33.78871
## 7         Asian_Indian            10   M       910   3333    27.30273
## 8         Asian_Indian            10   F       609   2070    29.42029
## 9         Asian_Indian            11   M       433   2055    21.07056
## 10        Asian_Indian            11   F       375   2009    18.66600
## 11    Not_Asian_Indian             7   M     31298 162254    19.28951
## 12    Not_Asian_Indian             7   F     14974  62619    23.91287
## 13    Not_Asian_Indian             8   M     88178 404792    21.78353
## 14    Not_Asian_Indian             8   F     43588 167672    25.99599
## 15    Not_Asian_Indian             9   M    141083 581588    24.25824
## 16    Not_Asian_Indian             9   F     75301 300274    25.07743
## 17    Not_Asian_Indian            10   M    142785 692364    20.62282
## 18    Not_Asian_Indian            10   F     95688 494749    19.34072
## 19    Not_Asian_Indian            11   M     94296 711185    13.25900
## 20    Not_Asian_Indian            11   F     99071 924464    10.71659
```

```
##       se_calc_1    se_calc_2         SE
## 1   2.222167969 0.3925500016 1.61700896
## 2  19.667672528 0.5320248183 4.49440734
## 3   1.202862387 0.3086308840 1.22942803
## 4   4.928002172 0.3990009897 2.30803015
## 5   0.789701083 0.2086718521 0.99918614
## 6   2.037517278 0.2895739032 1.52548064
## 7   0.595509735 0.1800611212 0.88066501
## 8   1.003128276 0.2173398354 1.10474799
## 9   0.809288311 0.2091759815 1.00918992
## 10  0.755689712 0.1761667775 0.96532714
## 11  0.009595238 0.0009843826 0.10285728
## 12  0.029056064 0.0013489185 0.17437024
## 13  0.004209152 0.0005726765 0.06915077
## 14  0.011473637 0.0008069101 0.11081763
## 15  0.003159215 0.0004973613 0.06046963
## 16  0.006257170 0.0005862345 0.08272487
## 17  0.002364336 0.0003976299 0.05255441
## 18  0.003153131 0.0003705148 0.05936030
## 19  0.001617158 0.0003235738 0.04405374
## 20  0.001034993 0.0001632936 0.03461627
```

```
    #Isolate SE column; to be added to tab1 for plotting graph
    SE <- SE %>% select(SE)
```

Transforming variables in tab1 for ease of plotting graph: Create new column **"Mid-decadal age group (Years)"** using existing Age_Recode_12; recode and relevel Sex and Asian-Indian Status to assist with order of the graphs/ legend key; and bind *Standard Error* column to plot the error bars.

```
#Create Mid-decadal age (years) column
tab1 <- tab1 %>% mutate(Mid_decadal_age = case_when(Age_Recode_12 == 7 ~ 50,
                                                    Age_Recode_12 == 8 ~ 60,
                                                    Age_Recode_12 == 9 ~ 70,
                                                    Age_Recode_12 == 10 ~ 80,
                                                    Age_Recode_12 == 11 ~ 90))
#Recode and relevel Sex
tab1$Sex <- fct_recode(tab1$Sex, Men = "M", Women = "F")
tab1$Sex <- fct_relevel(tab1$Sex, c("Women", "Men"))
#Recode and relevel Asian Indian Status
tab1$Asian_Indian_Status <- fct_recode(tab1$Asian_Indian_Status,
                                       "Asian Indian" = "Asian_Indian",
                                       "Not-Asian Indian" = "Not_Asian_Indian")
tab1$Asian_Indian_Status <- fct_relevel(tab1$Asian_Indian_Status,
                                        c("Asian Indian", "Not-Asian Indian"))
tab1 <- bind_cols(tab1, SE)
tab1
```

```
##    Asian_Indian_Status Age_Recode_12   Sex Percent_pop Lead_percent_pop
## 1         Asian Indian             7   Men    23.12500         8.955224
## 2         Asian Indian             7 Women    35.89744         7.014682
## 3         Asian Indian             8   Men    31.57016        12.756517
## 4         Asian Indian             8 Women    36.99789         9.604779
## 5         Asian Indian             9   Men    33.28592        12.456946
## 6         Asian Indian             9 Women    33.78871        12.569170
## 7         Asian Indian            10   Men    27.30273        13.123816
## 8         Asian Indian            10 Women    29.42029        13.667342
```

```
## 9          Asian Indian            11   Men    21.07056          9.247258
## 10         Asian Indian            11 Women    18.66600          9.802750
## 11     Not-Asian Indian             7   Men    19.28951          7.083545
## 12     Not-Asian Indian             7 Women    23.91287          6.847203
## 13     Not-Asian Indian             8   Men    21.78353          8.247833
## 14     Not-Asian Indian             8 Women    25.99599          8.341003
## 15     Not-Asian Indian             9   Men    24.25824          9.228914
## 16     Not-Asian Indian             9 Women    25.07743          9.040937
## 17     Not-Asian Indian            10   Men    20.62282          8.332813
## 18     Not-Asian Indian            10 Women    19.34072          8.183213
## 19     Not-Asian Indian            11   Men    13.25900          6.360413
## 20     Not-Asian Indian            11 Women    10.71659          5.717321
##    Excess_percent_deaths Mid_decadal_age         SE
## 1              14.169776              50 1.61700896
## 2              28.882754              50 4.49440734
## 3              18.813639              60 1.22942803
## 4              27.393106              60 2.30803015
## 5              20.828971              70 0.99918614
## 6              21.219537              70 1.52548064
## 7              14.178915              80 0.88066501
## 8              15.752948              80 1.10474799
## 9              11.823301              90 1.00918992
## 10              8.863253              90 0.96532714
## 11             12.205964              50 0.10285728
## 12             17.065667              50 0.17437024
## 13             13.535700              60 0.06915077
## 14             17.654989              60 0.11081763
## 15             15.029324              70 0.06046963
## 16             16.036493              70 0.08272487
## 17             12.290010              80 0.05255441
## 18             11.157503              80 0.05936030
## 19              6.898585              90 0.04405374
## 20              4.999267              90 0.03461627
```

Results

- In the entire population, there were **4,518,524 (22.43 %)** deaths due to contributing atherosclerotic-disease(s) and **2,017,221 (10.01 %)** deaths due to contributing diabetes mellitus. The number of atherosclerotic-disease(s) related deaths where diabetes mellitus also contributed were **830,865 (18.39 %)**.
- Cluster of atherosclerotic-disease(s) and diabetes mellitus as contributing causes correlated more strongly in Asian Indian **(Rho = 0.36, SE = 0.007)** as compared to Not Asian Indian **(Rho = 0.31, SE = 0.0005)**; difference between the groups: **p < 0.001**.
- The **graph** below shows that the excess fraction of deaths due to diabetes mellitus when atherosclerotic-disease(s) also contributed vs. when it did not, was higher in both AI men and women across all age groups in comparison to Not-AI but more so in younger AI women (age < 60y)

Graph

```
#Plot graph
plot <-
  ggplot(tab1,
```

```r
       aes(x = Mid_decadal_age, y = Excess_percent_deaths, color = Asian_Indian_Status)) +
scale_color_manual(values = c("#DC143C", "#1E90FF")) +
geom_line(size = 1.5) +    facet_wrap ( ~ Sex) +
#SE Bar
geom_errorbar(
  aes(ymin = Excess_percent_deaths - SE, ymax = Excess_percent_deaths + SE),
  width = 3,
  size = 1,
  position = position_dodge(0.1)
) +
geom_point(size = 2, color = "black") +
labs(
  title = "Excess Diabetes in Atherosclerotic Disease - Related vs. Unrelated Deaths",
  subtitle = "By Sex, Age, and Asian Indian Origin :-  United States (2012-2019)",
  x = "Mid-Decadal Age (Years)",
  y = "% Excess Deaths with Diabetes",
  caption = "Difference Percent +/- Standard Error Bars"
)  +
#Aesthetics
theme_igray() +
theme(
  plot.title = element_text(
    size = 24,
    face = "bold",
    color = "#660000",
    vjust = 2,
    hjust = 0.5,
    margin = margin(10, 0, 1, 0)
  ),
  plot.subtitle = element_text(
    size = 22,
    face = "bold",
    color = "#000000",
    vjust = 2,
    hjust = 0.5,
    margin = margin(10, 0, 1, 0)
  ),
  plot.caption = element_text(size = 15),
  axis.text.x = element_text(size = 18, face = "bold", vjust = 0.5),
  axis.text.y = element_text(size = 18, face = "bold", vjust = 0.5),
  axis.title.x = element_text(
    size = 20,
    face = "bold",
    color = "#000000",
    margin = margin(10, 0, 10, 0)
  ),
  axis.title.y = element_text(
    size = 20,
    face = "bold",
    color = "#000000" ,
    margin = margin(0, 10, 0, 10)
  ),
  strip.text.x = element_text(size = 14, color = "#000000", face = "bold"),
```

```
  strip.background = element_rect(
    color = "#696969",
    fill = "#E2DFAD",
    size = 1.5,
    linetype = "solid"
  ),
  plot.background = element_rect(fill = "#DFCAAF"),
  panel.border = element_rect(colour = "#000000", fill = NA)
) +
theme(
  legend.title = element_blank(),
  legend.background = element_rect(fill = "#FFFFFF"),
  legend.box.background = element_rect(colour = "#000000"),
  legend.text = element_text(size = "15")
)
#View plot
plot
```

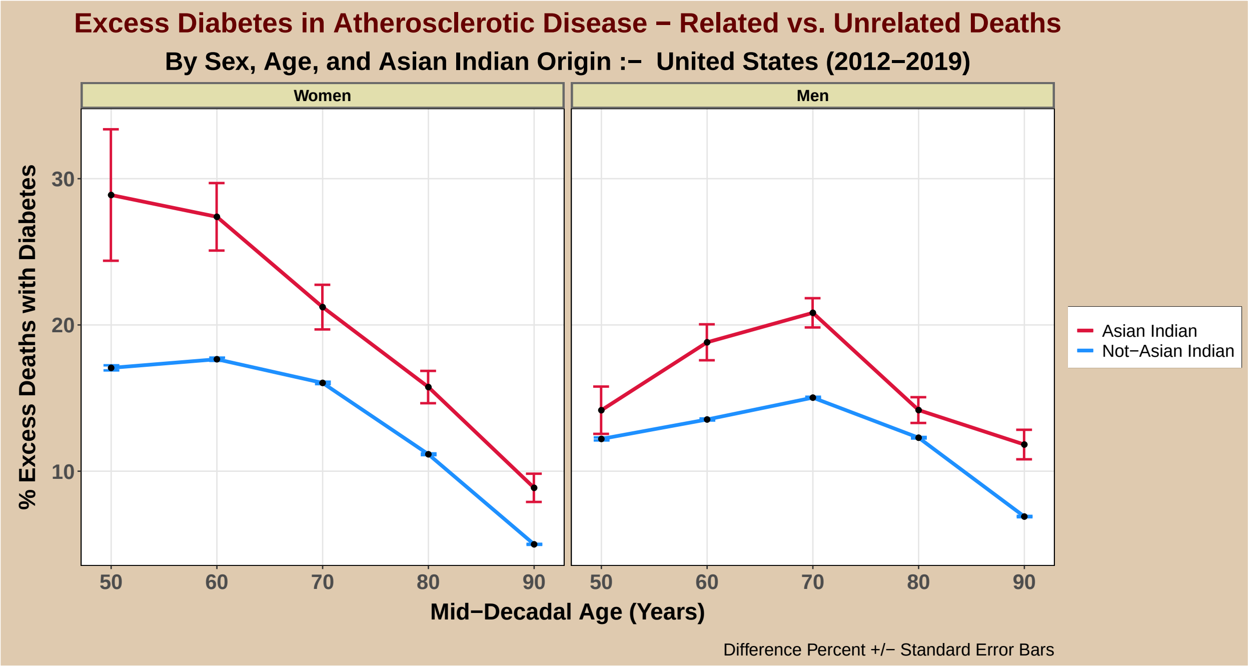

Check how the graph may appear to color-blind persons

```
cvdPlot(plot)
```

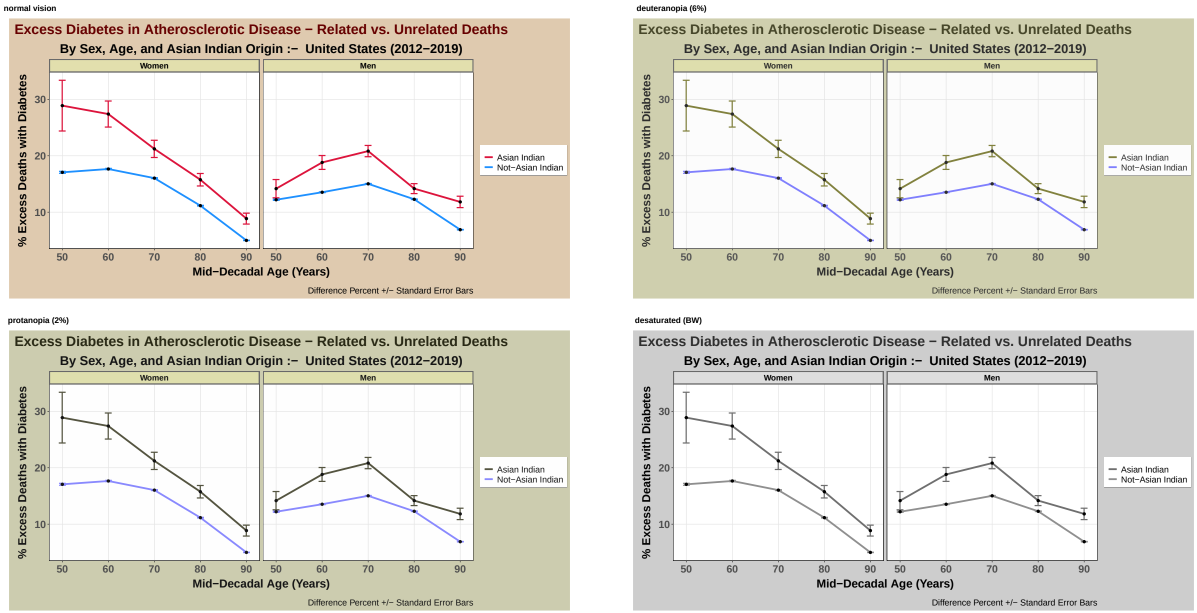


```r
tiff(
  "plot.tiff",
  units = "in",
  width = 16,
  height = 8,
  res = 900
)
plot
dev.off()
```
